# Household Air Pollution: Sources and Exposure Levels to Fine Particulate Matter in Nairobi Slums

**DOI:** 10.3390/toxics4030012

**Published:** 2016-07-13

**Authors:** Kanyiva Muindi, Elizabeth Kimani-Murage, Thaddaeus Egondi, Joacim Rocklov, Nawi Ng

**Affiliations:** 1 African Population and Health Research Center (APHRC), P.O. Box 10787-00100 Nairobi, Kenya; ekimani@aphrc.org; 2 Epidemiology and Global Health Unit, Department of Public Health and Clinical Medicine, Faculty of Medicine, Umeå University, Umeå SE-901 87, Sweden; joacim.rocklov@umu.se (J.R.); nawi.ng@umu.se (N.N.); 3 Drugs for Neglected Diseases Initiative, P.O. Box 21936-00505 Nairobi, Kenya; tegondi@gmail.com

**Keywords:** household air pollution, cookstoves, PM_2.5_, slums, Nairobi

## Abstract

With 2.8 billion biomass users globally, household air pollution remains a public health threat in many low- and middle-income countries. However, little evidence on pollution levels and health effects exists in low-income settings, especially slums. This study assesses the levels and sources of household air pollution in the urban slums of Nairobi. This cross-sectional study was embedded in a prospective cohort of pregnant women living in two slum areas—Korogocho and Viwandani—in Nairobi. Data on fuel and stove types and ventilation use come from 1058 households, while air quality data based on the particulate matters (PM_2.5_) level were collected in a sub-sample of 72 households using the DustTrak™ II Model 8532 monitor. We measured PM_2.5_ levels mainly during daytime and using sources of indoor air pollutions. The majority of the households used kerosene (69.7%) as a cooking fuel. In households where air quality was monitored, the mean PM_2.5_ levels were high and varied widely, especially during the evenings (124.6 µg/m^3^ SD: 372.7 in Korogocho and 82.2 µg/m^3^ SD: 249.9 in Viwandani), and in households using charcoal (126.5 µg/m^3^ SD: 434.7 in Korogocho and 75.7 µg/m^3^ SD: 323.0 in Viwandani). Overall, the mean PM_2.5_ levels measured within homes at both sites (Korogocho = 108.9 µg/m^3^ SD: 371.2; Viwandani = 59.3 µg/m^3^ SD: 234.1) were high. Residents of the two slums are exposed to high levels of PM_2.5_ in their homes. We recommend interventions, especially those focusing on clean cookstoves and lighting fuels to mitigate indoor levels of fine particles.

## 1. Introduction

Air pollution is one of the most serious environmental risks to human health [1]. In 2012, air pollution was responsible for an estimated seven million premature deaths globally [2]. Household air pollution accounted for 4.3 million of these deaths [3]. Particulate matter with an aerodynamic diameter of 2.5 microns and below (PM_2.5_) has been singled out as a pollutant of great concern, owing to its link with adverse effects on human health [4,5,6]. Globally, it is estimated that 2.8 billion people, mainly those living in poor households in low- and middle-income countries (LMICs), use biomass fuels for cooking and heating [7]. The contribution of particulate matter to poor indoor air quality, resulting from the combustion of biomass fuels, has been documented [8,9,10,11]. Further, evidence demonstrates that there is a relationship between health outcomes and exposure to these emissions [4,12,13,14]. There is evidence showing that exposure to air pollution is associated with adverse pregnancy outcomes, such as low birth weight [15,16,17], pre-term births [18,19] and still births [16,17,20]. 

The air pollution health discourse has been dominated by evidence from countries outside of Africa [12,21,22,23], despite the high proportion of African households that are dependent on biomass fuels [24]. Outdoor concentrations of pollutants, which greatly influence indoor concentrations, have remained high in the region, especially in urban areas [25,26], exceeding the 24 h mean of 25 µg/m^3^ according to the World Health Organization (WHO) guidelines. A few studies measuring levels of household air pollution have been conducted in sub-Saharan Africa (SSA), including Kenya. These have typically focused on households in rural areas, where biomass fuel predominates [11,27,28,29]. Therefore, the unique challenges confronting poor urban neighborhoods and slums have remained unexamined. Slum residents might face higher concentrations of pollutants in homes because of the high population density living in single-room houses with poor ventilation. This crowding may lead to a ‘neighborhood effect’ on indoor air quality, where neighboring households may influence other households’ air quality due to indoor emissions. Indoor air quality may also be affected by outdoor levels of pollution, particularly in areas located near outdoor emission sources such as industries, dumpsites or high-traffic roads. Slum households also face other barriers that might compound their exposure to higher concentrations of indoor pollutants; insecurity (i.e., criminal activities such as robbery), for example, has been reported as one reason to limit the use of ventilation in slum dwellings. Widespread poverty also prevents a household’s ability to move up the fuel ladder, restricting it to the use of polluting fuels including unorthodox fuels such as plastics [30].

Given the implications of household air pollution for health, this study examines the indoor environment of households in communities facing environmental and economic challenges. Specifically, the study aims to describe the fuels and stove types used by households in slums, and the availability and use of ventilation in these houses. In addition, we characterize the variation of household PM_2.5_ concentrations by type of cookstove. 

## 2. Materials and Methods 

### 2.1. Study Setting

This study was conducted in the Korogocho and Viwandani slums of Nairobi, which form the Nairobi Urban Health and Demographic Surveillance System (NUHDSS) study areas. The NUHDSS is a longitudinal platform that collects key demographic data every four months from all households within the study area. The platform also provides an ideal sampling frame for supplementary nested studies. The two communities exhibit some differences in important socio-demographic factors. While Korogocho is poorer economically, Viwandani is composed of workers from the nearby industrial zone. In addition, Korogocho has overall lower educational attainment compared with Viwandani. An important feature of the study sites with a bearing on this study is the nature of houses and living arrangements. Most households occupy a single room used as the kitchen and bedroom, and the main material used to construct these houses’ walls and roofs is zinc sheeting. Data from the NUHDSS indicate that more than 90% of households cook in the room used as a living and sleeping room [31], with implications for indoor air quality. Further, an earlier study nested in the NUHDSS showed that residents in the two slums face extremely high concentrations of outdoor fine particles. This is mostly due to the burning of waste in dumpsites and on streets, biomass use and industrial emissions, as well as re-suspension of dust from unpaved roads [32]. A detailed description of the NUHDSS can be found here [33,34]. 

### 2.2. Study Design Sample and Data Collection

This study on household air pollution was nested in a randomized controlled intervention study of pregnant women receiving pre- and post-natal counseling on nutrition to ensure better health outcomes for mothers, infants and children in the two NUHDSS slums in 2012–2014 [35]. The intervention study recruited pregnant women from approximately 1418 households out of the 28,000 NUHDSS households during the baseline survey conducted in 2012–2013. Of the 1418 households in which a pregnant woman resided at baseline, 1058 gave complete interviews for the air pollution module and are the basis of our analysis on fuel and stove types, ventilation use and exposure to tobacco smoke, while PM_2.5_ data came from 72 conveniently selected households. 

#### 2.2.1. Questionnaire-Based Data Collection

For the households recruited into the intervention study, the following data were collected: socio-demographic information (including mother’s age, marital status, mother’s education level, and employment status and the nature of place they work), types of cooking stoves and fuels used, frequency of cooking and doing other household chores, availability and use of ventilation at home, women’s smoking status and their exposure to second-hand cigarette smoke at home and at the work place for those who worked. Data were collected by a team of well-trained female interviewers who were preferred culturally for a study among pregnant women. Data were collected using questionnaires administrated through electronic devices. Data quality was assured through internal checks on the questionnaire; team leaders also checked all completed work for errors before sending the data for cleaning. Wealth scores were generated for all NUHDSS households using principal component analysis of household possessions and amenities, collected through NUHDSS’ annual update round. These scores were then used to generate wealth categories for households in the study area, which were later linked to the data in this study. 

#### 2.2.2. Particulate Matter Measurement

We obtained a list of households participating in the intervention study with information on the main fuel used for cooking as reported in the NUHDSS data. We took a convenient sample of at least 25 households in each slum to represent households using different types of cooking fuels. In Viwandani, an additional 23 households were monitored, bringing the total to 73 households from both communities. One household was dropped from the Korogocho sample owing to a short monitoring duration of less than one hour, leaving a total of 72 households in the analytical sample. A convenient sample was necessitated by concerns for equipment safety as well as availability of a household member for most of the day, to facilitate retrieval of equipment for re-charging of batteries especially in households without electricity. 

Household PM_2.5_ levels were monitored using DustTrak™ II Model 8532 (TSI Inc., Shoreview, MN, USA) monitors. Field staff checked impaction plates daily before placing them in dwellings, to ensure the monitors were properly oiled. Daily zeroing of the monitors was done before monitoring commenced, and data collection teams received training on how to check for error messages and change filters when prompted. Monitors were placed in the cooking area at a level equivalent to the breathing zone of an individual engaged in cooking, and data were logged every minute. At the time of placing the monitor, the field staff noted the room identifier and the type of fuel that was in use if cooking was in progress. Where no cooking was taking place, the staff asked about the type of fuel to be used for most of the household’s cooking. Each home was monitored only once due to limited number of monitors (one per site). Monitors were left in the house as long as was practically possible to capture concentrations both when the stove was and was not in use. On average, households were monitored for 10.4 h in Viwandani and 11.8 h in Korogocho. Data were collected between May and October 2014, which provided an opportunity to monitor air quality during wet and dry seasons when stove and ventilation use may vary. Data were sent daily to the lead researcher who checked for possible errors such as negative readings or flow errors. Constant communication between research and data collection teams allowed for swift resolution of any issues. Equipment was shipped to the manufacturer at the start of 2014 for scheduled calibration, before the start of the PM_2.5_ monitoring study. Fieldwork was interrupted for some weeks in July–August due to monitor malfunction and subsequent repairs. 

#### 2.2.3. Calibration of PM_2.5_ Measurements

Fine particle data were corrected for over-estimation using a calibration factor computed using the following formula:

Calibration Factor = (Gravimetric Concentration)/(DustTrak Concentration)(1)

The calibration factor was computed after simultaneous particle collection within the study area, using gravimetric personal monitors (BGI model 400, Mesa Labs Inc., Butler, NJ, USA) alongside the DustTrak™. We worked with the Institute of Nuclear Science and Technology at the University of Nairobi, which provided gravimetric monitors and a micro-balance for weighing the filters before and after particle collection in the Institute’s laboratory. Standard procedures were followed in the collection and handling of samples using the gravimetric monitor to ensure there was no undue influence of temperature and humidity on the samples. Further details on the calibration exercise can be found in previous work [32]. Sample collection for each run was only possible during daytime hours and was done for a total of 24 h over three days in both study communities (which explains the variation in mass in the two samples). All samples were collected outdoors. The results are shown in Table 1 below. The correction was necessitated by the fact that the DustTrak™ monitors have been factory calibrated using Arizona test dust and have been found to over-estimate PM_2.5_ levels [36]. We applied the calibration factor to each one-minute mass of fine particles. 

### 2.3. Statistical Analysis

We compared the types of fuel and stoves used and the patterns of ventilation use in participants’ households in the two slum areas in Nairobi. We analyzed the differences in the distribution observed between the two slum areas using the Chi-square test. In addition, we described women’s exposure to tobacco smoke. For the analysis of levels of PM_2.5_, we restricted our analysis to 70 of all 72 households measured, where monitoring was done for at least four hours. We assessed the variation of PM_2.5_ levels across households using different fuel types.

### 2.4. Ethical Consideration

The intervention study in which this study was nested was granted ethical clearance by the Kenya Medical Research Institute’s Ethics Review Committee (Identification code: Protocol No. 327; date of approval: 2 November 2012). The air pollution study was granted ethical clearance by the Ethics and Scientific Review Committee at Amref Health Africa. We sought individual informed consent from respondents in households participating in the study. In addition, permission to place our monitors within sampled houses was obtained from the respondent or an adult member of the household.

## 3. Results

### 3.1. Fuels, Stoves and Ventilation

Most households reported using different combinations of stove types and cooking fuels with the kerosene stove reported as the most commonly used stove (69.7%). However, there was considerable use of other different stoves and fuels during the four-month period preceding the survey. Regarding the availability of ventilation, 37.6% of the houses in the study area had at least one door and no window(s). These household characteristics are summarized in Table 2.

**Figure 1:**
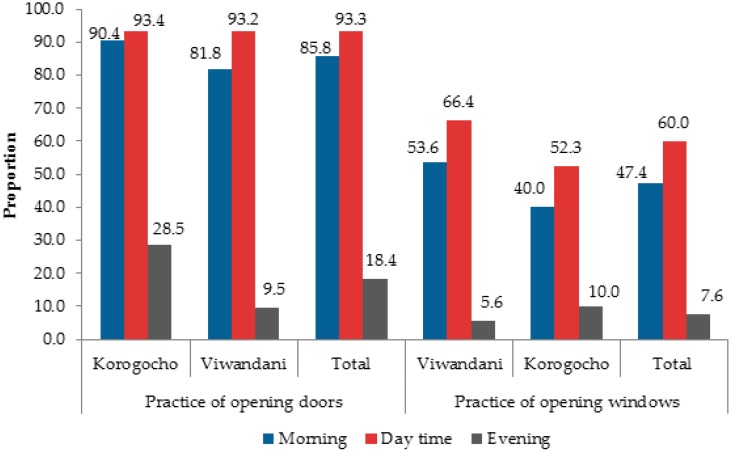
Proportion (%) of households opening doors and windows during cooking times.

When we assessed the patterns of use of windows for ventilation among households reporting to have a window, we observed that, generally, few households opened windows when preparing meals. Only 47.4% of the households reported opening windows in the morning, 60.0% during the day, and 7.6% in the evening. A large proportion of households opened their doors during cooking in the morning (85.8%) and during the day (93.3%), while only a small percentage (18.4%) of households reported cooking with their doors opened during the evening (see Figure 1). The results indicated that in the evenings, most households, especially in Viwandani, cooked with the doors and windows closed. This was also the time that most household members were within the house after returning from their day’s activities. 

### 3.2. Exposure to Tobacco Smoke 

Very few respondents reported having smoked at any point in their lives (2.5%; *n* = 26) or being current smokers (less than 1%; *n* = 8). However, 14.7% reported that one or more members of their household smoked, with 6.7% smoking inside the house. Another 3.2% were exposed to tobacco smoke at work. 

### 3.3. Household Air Quality: PM_2.5_ Levels

**Figure 2:**
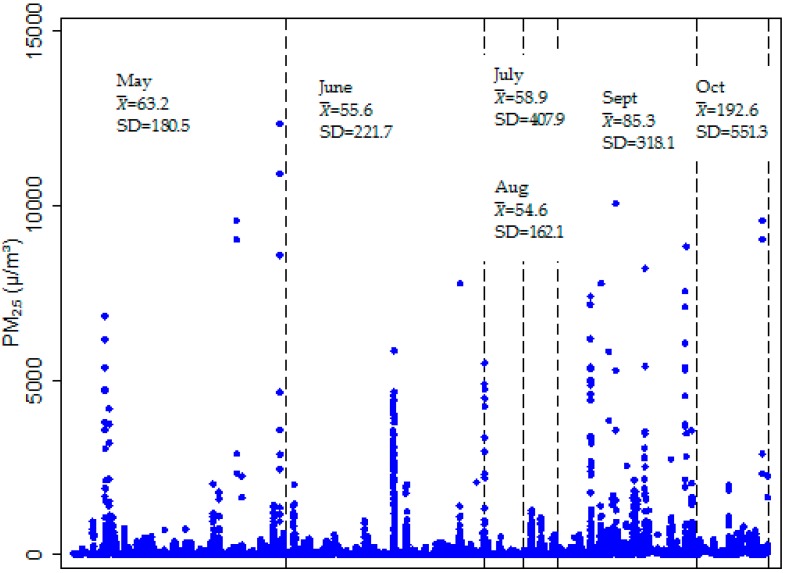
One minute average indoor PM_2.5_ concentrations for the monitoring period.

We present the PM_2.5_ concentrations in the 72 households monitored for at least four hours. PM_2.5_ levels varied widely over the monitoring duration ranging from 1 to 12,369 µg/m^3^ (SD = 287.11). Although the monitoring duration was below 24 h, we noted that mean levels were below the WHO 24 h limit of 25 µg/m^3^ in 13.7% of households, while the rest of the households exceeded this limit. Further, 18.0% of households had levels of PM_2.5_ at or above 100 µg/m^3^, with the highest mean recorded at 261.8 µg/m^3^. The data showed peaks in indoor concentrations in May, September and October (Figure 2).

#### 3.3.1. PM_2.5_ Concentrations by Main Fuel Used for Cooking and Lighting

Table 3 displays the distribution of households by fuel type and levels of PM_2.5_. The fuel types presented here are for households in which PM_2.5_ monitoring was conducted. The results indicate that charcoal/wood was the most commonly used fuel in Korogocho while kerosene dominated in Viwandani. There was also concurrent use of different fuels and stoves in the monitored households, particularly among those who relied primarily on kerosene and Liquefied Petroleum Gas (LPG) (data not shown). Most of these households simultaneously used charcoal, a preferred fuel for preparing some traditional dishes and those that take more time to cook, since it is cheaper compared to LPG and kerosene. 

**Figure 3:**
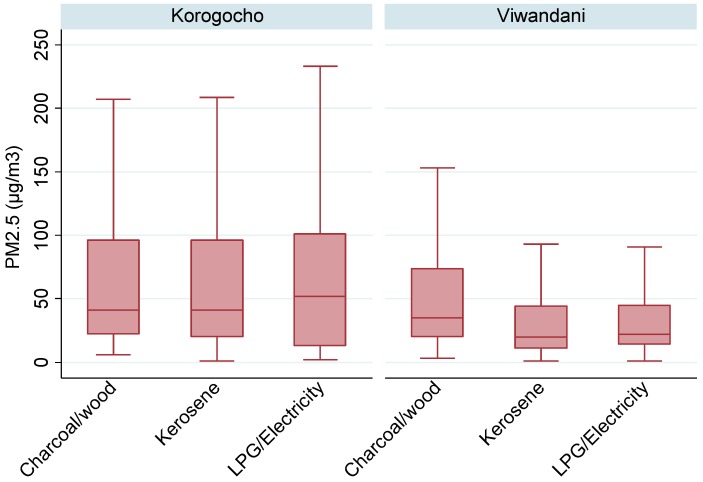
Box plot of PM_2.5_ associated with different cooking fuels.

We observed variations in the PM_2.5_ levels across the study communities, with households in Korogocho having higher PM_2.5_ levels than in Viwandani (see Figure 3). Households using kerosene also had PM_2.5_ levels almost equal to those observed in charcoal/wood-using households. Overall, all fuel types were associated with elevated levels of PM_2.5_.

The box plot of PM_2.5_ levels in Figure 3 (which excludes outliers) indicates an almost similar spread of concentrations in Korogocho, especially in charcoal- and kerosene-using households, while those for households using LPG/electricity were quite spread out. On the other hand, concentrations in Viwandani homes were not as spread out as those in Korogocho. The median levels in all households, except those reliant on LPG/electricity in Korogocho, were below 50 µg/m^3^. 

Household sources of light can have a significant impact on indoor levels of pollutants, especially if they are open flames, such as candles or kerosene lamps. In Kenya, most households without access to electricity rely on a simple kerosene tin lamp locally known as “koroboi”. Our results (Figure S1) indicate that households using kerosene lamps for lighting had extremely high PM_2.5_ levels (181.7 µg/m^3^), and are likely one of the reasons why evening emissions were higher than daytime emissions in both study sites. 

#### 3.3.2. PM_2.5_ Concentrations at Different Times of the Day

**Figure 4:**
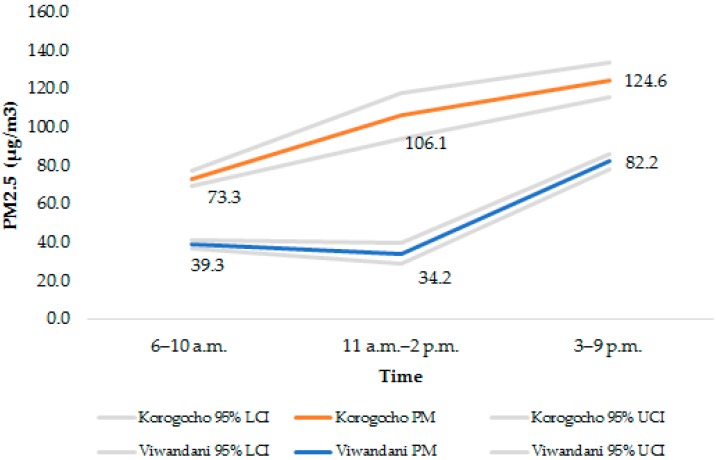
Mean PM_2.5_ concentrations at different cooking times.

We categorized the monitoring time into periods covering the monitoring duration in a given day to overlap meal preparation periods (see Figure 4). We noted a relatively higher concentration in Korogocho from morning until the evening compared to Viwandani (ranging from 73.3 to 124.6 µg/m^3^ compared to 39.2–82.2 µg/m^3^). The PM_2.5_ level was slightly lower in Viwandani over the period between 11 a.m. and 2 p.m. The levels recorded in Viwandani sharply increased in the evening (3 p.m.–9 p.m.) to a concentration of 82.2 µg/m^3^. Only one household was monitored overnight and the mean PM_2.5_ recorded after 9 p.m. until 5 a.m. was 48.2 µg/m^3^.

## 4. Discussion

This paper describes household air pollution in two Nairobi urban slums. We found that a considerable proportion of houses had no windows and were therefore forced to rely on the door as a main route to vent indoor emissions. In addition, a large proportion of households did not open windows and/or doors during cooking, especially in the evening, which has implications for higher pollutant concentrations. This finding resonates with a studies in two sub-Saharan African countries, Ghana and Malawi, which found levels of fine particles to be notably high in kitchens [26,29]. The elevated levels of fine particles during certain times have been associated with increased individual exposure, and, consequently, poor health.

The lack of windows in some houses is a worrying structural omission, which points to an unregulated slum “real estate” and also points to the vulnerability of occupants of such houses to high concentrations of indoor pollutants. The finding that despite having windows, some people chose not to open them even during cooking times can be explained by findings from a qualitative study that attributed this to structural limitations, security and privacy concerns within slum settings [30]. These deterrents to opening windows appear strong enough to limit the use of this ventilation avenue in the two communities. The decision not to open windows might also point to gaps in knowledge about cookstove emissions and their effects on health, and the role that ventilation can play in reducing indoor concentrations of pollutants.

A considerable number of households prepared food with both windows and doors closed, especially in the evening when the majority of the members are in the dwelling, further increasing exposure to emissions from cookstoves. Lamps are also more widely used in the evening, and the reliance on kerosene for lighting, especially the open-wick lamp, is a major contributor of indoor fine particles [37,38] and poor health, as has been reported elsewhere [39,40]. A previous qualitative study in the area indicates that residents have blocked the eaves spaces to keep out the polluted outdoor air and nuisances, e.g., mosquitoes [30]; one can picture the polluted spaces household members live in when all major ventilation is unavailable. Further, the evening was said to be the time when fires were lit on the dumpsites in the vicinity of both communities, while industries were said to release most emissions during this time. These outdoor sources would have an impact on indoor levels, especially in areas closest to them. Indeed, results indicate that levels of PM_2.5_ are highest in the evening, perhaps arising from a lack of ‘escape’ routes for stove and lamp emissions. These concentrations, especially those observed during the evening, were higher than the 8 h outdoor concentrations reported for Viwandani (67 μg/m^3^) [32]. 

There was evidence of the simultaneous use of different cooking fuels and stoves in all households surveyed, as has been found in other surveys [41,42]. Most households relied on kerosene stoves but they also reported using other types of cookstoves, mainly charcoal stoves. Qualitative findings indicate that economic challenges have pushed some households in these communities to use crude fuels such as cloth and plastic waste [30], a factor that has also been reported to drive the types of fuels used elsewhere [41,43]. 

Besides cookstoves and lighting fuels, household air quality is affected by other behaviors from household members. Key among these behaviors is cigarette smoking within the house which has an impact on the levels of PM_2.5_. For example, a study in Scottish homes found levels of PM_2.5_ in households with a member who smoked indoors that were 10 times higher than in homes without a smoker, and three times the annual limit for exposure to PM_2.5_ [44]. In Kenya, the prevalence of cigarette smoking among adolescents is, respectively, 13% and 7% among boys and girls [45], while among adults it is estimated to be 11% [46]. National estimates indicate that 14.3% of people are exposed to second-hand tobacco smoke at home [47]. In a context where patriarchy is the dominant familial structure and where masculine norms have been associated with cigarette smoking [48], it is not surprising that members of households (mostly male) would smoke within the house [49]. Given emerging evidence that fine particles from tobacco smoke linger for hours before dropping to the WHO guideline levels [50], household members including the unborn fetus [51,52,53] remain exposed to health-damaging levels even after smoking has ceased.

The results presented should be interpreted in light of the following limitations. First, as we embedded this study in an on-going study among pregnant women, we only conducted this study in households with pregnant women. We, however, have no reason to believe that these households differ from other households in general. Secondly, we were unable to conduct continuous 24 h monitoring. This was due to concerns about the safety of data collectors as well as the equipment if left overnight in the sampled households. Even though the results from this study are not directly comparable with other studies following the WHO guidelines, the levels of particulate matter measured in less than 24 h indicate elevated levels within homes in the two communities. Therefore, we argue that the existing evidence, though not representative of a 24 h measurement, is sufficient to initiate actions to mitigate this important public health problem. Thirdly, we did not collect information regarding times when indoor smoking took place in the sampled homes, which is an important behavior that contributes to high levels of PM_2.5_. Fourthly, the household sample selected for PM_2.5_ monitoring was small and drawn conveniently from a random sample of households, purely due to the technical reasons described above. Lastly, we did not correct the measurements for meteorological variables such as humidity and temperature, which might have an impact on the levels of fine particles. The DustTrak^®^ monitors did not have an inbuilt monitoring capability for these variables, hence these data were not available. However, weather data for Nairobi indicate that during the monitoring period, the mean temperature ranged from 18.3 to 21.4 °C and relative humidity ranged from 43% to 59%, making it less prone to overestimate the PM_2.5_ level as would have occurred with high humidity over 80%. Despite these limitations, we are confident that the results still remain generalizable since these conveniently sampled households relied on similar fuels and stoves for cooking as other households in the study areas. 

Despite the limitations, this study provides evidence of the levels of indoor air pollution experienced by populations in two urban slums in Nairobi. The study can contribute to the air quality policy discourse in Kenya. It demonstrates the importance of developing intervention programs to minimize emissions and educate the population on the gains of using cleaner cooking and lighting systems. This would call for government intervention in making cleaner fuels and stoves more available to the population at an affordable cost. Educating the population on the importance of ventilation use when cooking, and promoting behavior change, such as the consistent use of available ventilation during stove use, will certainly be necessary coupled with social changes to ensure security in the communities. The concern about security has been cited as a deterrent to the use of ventilation, especially in the evenings [30]. Further insecurity has been implicated in curtailing other important activities including health care service-seeking [54] and schooling [55]. Therefore, addressing insecurity in these settings might result in the improvement of health and social outcomes in the population.

## 5. Conclusions 

We conclude that people residing in the two slum communities in Nairobi are exposed to high levels of fine particulate matter within their homes, mainly from cooking and lighting fuels and also the smoking of cigarettes indoors. Based on these findings, we recommend community campaigns to create awareness of household air pollution which could include a new approach of personalized feedback on levels of pollutants [56] to encourage behavior change, especially those related to proper ventilation and indoor smoking cessation. Interventions aiming to reduce household air pollution in these settings, particularly those focusing on the improvement of cooking and lighting systems and behavior change promotion to ensure adoption and consistent use of these systems, would be timely. Further study with 24 h measurements of pollutant concentrations is warranted.

## Figures and Tables

**Table 1:** PM_2.5_ readings from gravimetric and DustTrak monitors.

Date	Filter	Concentration (µg/m^3^)		Calibration Factor
		DustTrak	BGI 400	
13–15 February 2013	February 129	115	43	0.37
16–20 February 2013	February 130	125	31	0.25
Average Calibration Factor				0.31

**Table 2:** Distribution of ventilation type and cookstoves in the study areas.

Household Characteristics	Korogocho (%) (*n* = 499)	Viwandani (%) (*n* = 559)	Total (%) (*n* = 1058)	χ^2^ (*p*-Value)
**Ventilation type**				2.06 (*p* = 0.15)
Door and window	60.1	64.4	62.4	
Door only	39.9	35.6	37.6	
**Commonly used Cookstoves**				40.88 (*p* < 0.001)
Kerosene	71.7	67.8	69.7	
Charcoal/wood	26.1	19.9	22.8	
Gas/Electricity	2.2	12.3	7.6	
**Range of stoves used**				NA
Kerosene stove	94.0	93.9	94.0	
Metal/ceramic jiko	76.2	73.2	74.6	
Gas/electric stove	3.6	18.4	11.4	
Traditional 3-stone	1.0	0.2	0.6	

Note: The Chi-square for “range of stoves used” could not be calculated as multiple responses were allowed for this question.

**Table 3:** Distribution of households by fuel type and mean PM_2.5_ levels.

Outcome	Korogocho (24)	Viwandani (48)	Total	Test Statistic (*p*-Value)
				χ^2^ (*p*-value)
**Proportion of households using different cooking fuels (%)**				24.6 (*p* < 0.001)
Charcoal or wood	62.5	14.6	30.6	
Kerosene	12.5	72.9	52.8	
LPG/electricity	25.0	12.5	16.7	
**PM_2.5_ mean levels for different cooking fuels (µg/m^3^)**				*t*-statistic (*p* value)
Charcoal or wood	126.5	75.7	110.0	6.59 (*p* < 0.001)
Kerosene	109.6	58.7	61.9	7.43 (*p* < 0.001)
LPG/electricity	72.0	45.6	59.1	10.04 (*p* < 0.001)

## Supplementary Materials

The following are available online at www.mdpi.com/2305-6304/4/3/12/s1, Figure S1: Mean PM_2.5_ levels associated with household lighting fuels.
