# Supplementary material for: Household Air Pollution: Sources and Exposure Levels to Fine Particulate Matter in Nairobi Slums
Source: Toxics. 2016 Jul 13;4(3):12. doi: 10.3390/toxics4030012 (PMC5606663; doi:10.3390/toxics4030012)
Supplement: Supplementary file 1 [file toxics-04-00012-s001.pdf]

Supplementary Materials: Household Air Pollution: Sources and Exposure Levels to Fine Particulate Matter in Nairobi Slums

Kanyiva Muindi, Elizabeth Kimani-Murage, Thaddaeus Egondi, Joacim Rocklov and Nawi Ng

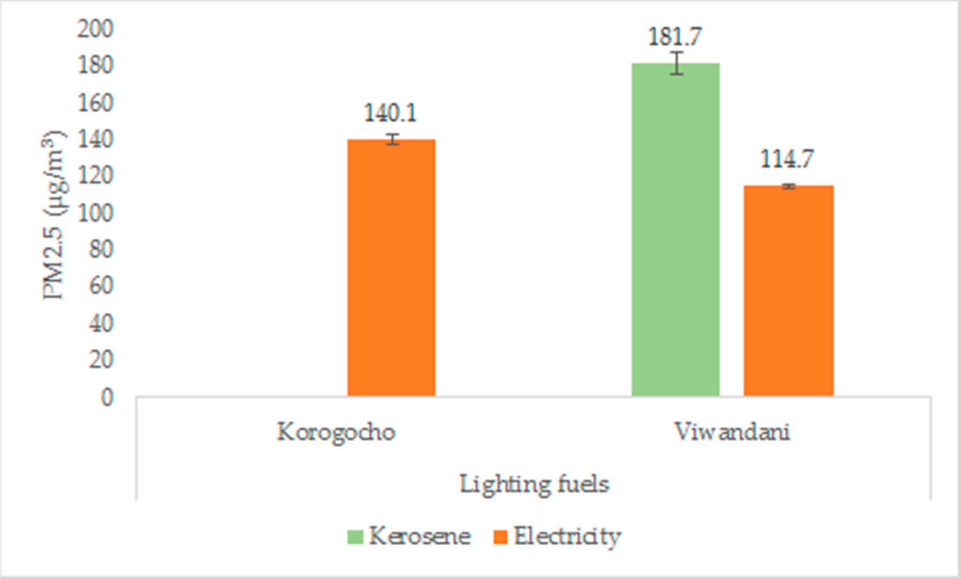

Figure S1. Mean $PM_{2.5}$ levels associated with household lighting fuels.

© 2016 by the authors. Submitted for possible open access publication under the terms and conditions of the Creative Commons Attribution (CC-BY) license (http://creativecommons.org/licenses/by/4.0/).
